# Premature termination of DNA Damage Repair by 3-Methyladenine potentiates cisplatin cytotoxicity in nasopharyngeal carcinoma cells

**DOI:** 10.1371/journal.pone.0329272

**Published:** 2025-08-04

**Authors:** Jie Zhou, Sisi Liu, Jiali Deng, Longmei He, Binyuan Jiang

**Affiliations:** 1 Department of Clinical Laboratory, The Affiliated Changsha Central Hospital, Hengyang Medical School, University of South China, Changsha, Hunan, China; 2 Medical Research Center, The Affiliated Changsha Central Hospital, Hengyang Medical School, University of South China, Changsha, Hunan, China; 3 Clinical Trials Office, The Affiliated Changsha Central Hospital, Hengyang Medical School, University of South China, Changsha, Hunan, China; National Institute of Cancer Research, TAIWAN

## Abstract

3-Methyladenine (3-MA) is widely recognized as a PI3K inhibitor involved in autophagy regulation. However, it is also a byproduct of DNA damage repair, and its role in modulating DNA damage response (DDR) mechanisms remains largely unexplored. Cisplatin (CDDP), a cornerstone chemotherapeutic agent for nasopharyngeal carcinoma (NPC), exerts its cytotoxic effects by inducing DNA damage in tumor cells. This study investigates the combined effects of CDDP and 3-MA on NPC cells. Cell viability and the half-maximal inhibitory concentration (IC50) were assessed using the Cell Counting Kit-8 (CCK-8) assay. Flow cytometry was employed to analyze cell cycle distribution, mitochondrial membrane potential (MMP) alterations, and apoptosis. γ-H2AX foci formation and morphological changes were examined via fluorescence microscopy, while Western blotting was used to evaluate proteins associated with the DNA damage response. The combination treatment significantly reduced cell viability and lowered the IC50 compared to CDDP alone. While both treatments induced Sub-G1 phase arrest, the combination resulted in greater MMP loss and apoptosis. Western blot analysis further revealed that 3-MA enhanced CDDP cytotoxicity by suppressing ATM/ATR/p53-mediated DNA damage repair and promoting apoptotic signaling. These findings suggest that 3-MA sensitizes NPC cells to CDDP by disrupting DNA repair processes, offering a promising therapeutic strategy.

## Introduction

Nasopharyngeal carcinoma (NPC) is a type of epithelial cell-derived tumor that occurs in the nasopharynx. The incidence of NPC exhibits a distinct geographical distribution, with a higher prevalence in North Africa and Southeast Asia. Studies have identified several risk factors for NPC, including genetic predisposition, climate, dietary habits, and viral infections [1]. Currently, the primary treatment options for NPC are surgery and radiochemotherapy. Early-stage NPC can be effectively treated with surgery, while advanced-stage NPC requires the addition of radiotherapy and chemotherapy [2].

Cisplatin (CDDP) is a first-line chemotherapeutic agent for nasopharyngeal carcinoma (NPC), widely used for its ability to enhance tumor radiosensitivity and improve treatment outcomes. However, its clinical utility is constrained by severe adverse effects, including nephrotoxicity, neurotoxicity, and ototoxicity [3,4]. The development and complexity of tumor diseases are closely associated with cisplatin resistance, which may arise from aberrant DNA damage repair mechanisms in tumor cells, the presence of cancer stem cells, and the remodeling of the extracellular matrix during tumor cell evolution and adaptation [5,6]. These factors severely compromise the efficacy of treatment.

Cells respond to cisplatin-induced DNA damage through multiple repair pathways, including base excision repair (BER), nucleotide excision repair (NER), homologous recombination (HR), and non-homologous end joining (NHEJ). BER handles non-bulky DNA base damage, NER manages bulky lesions, both are the primary pathway responsible for repairing cisplatin-induced DNA damage, while HR and NHEJ facilitate the repair of double-strand breaks [7,8]. The activation and coordination of these repair pathways rely on a complex network of proteins and signaling cascades. Central to this process are the ATM and ATR kinases, which serve as damage sensors that phosphorylate downstream effectors. ATR phosphorylation at Ser428 and ATM phosphorylation at Ser1981 initiate the DDR cascade, leading to cell cycle arrest and DNA repair [9].

The tumor suppressor protein p53 is a key regulator of the DDR. Phosphorylation of p53 at Ser15 stabilizes the protein and promotes the transcription of DNA repair-associated genes. However, when DNA damage is irreparable, p53 phosphorylation at Ser15 decreases, leading to total p53 degradation. In contrast, phosphorylation at Ser46 promotes apoptotic signaling. The efficiency of these DDR mechanisms is a critical determinant of cellular sensitivity to cisplatin, with DDR deficiencies enhancing drug sensitivity and excessive repair mechanisms contributing to resistance [10,11].

3-Methyladenine (3-MA) is a small-molecule inhibitor primarily known for its role in autophagy inhibition. Previous studies have demonstrated that combining 3-MA with cell division-related agents, such as paclitaxel or polo-like kinase 1 (PLK1) inhibitors, enhances cytotoxicity in NPC cells [12]. However, due to its dual role in autophagy regulation—suppressing autophagy under nutrient-rich conditions while promoting it under nutrient deprivation [13]—the precise mechanism underlying its synergy with paclitaxel remains unclear. Notably, 3-MA is also a byproduct of DNA alkylation damage repair [14,15], suggesting a potential link between 3-MA and DDR modulation. Based on this, we hypothesized that 3-MA may potentiate the effects of cisplatin by interfering with DDR pathways.

In this study, we observed that 3-MA significantly enhanced CDDP-induced apoptosis in NPC cells, accompanied by the upregulation of apoptosis-related proteins. We further investigated the molecular mechanisms underlying this synergistic effect, focusing on the role of 3-MA in modulating DDR pathways and its potential to sensitize NPC cells to cisplatin-induced cytotoxicity.

## Materials and methods

### Cell culture and drugs

The human NPC cell lines 5-8F (characterized by high tumorigenic and metastatic potential) and 6-10B (characterized by high tumorigenic but low metastatic potential) derived from the SUNE1 cell line and were obtained from the Cancer Research Institute of Central South University. Cells were cultured in a humidified incubator maintained at 37°C with 5% CO₂. The complete medium consists of DMEM medium (Biological Industries), 10% fetal bovine serum (FBS; Biological Industries), 100 U/mL penicillin, and 100 μg/mL streptomycin (Biological Industries). 3-MA was procured from MedChemExpress and dissolved in double-distilled water (ddH₂O), while CDDP was obtained from APExBIO and dissolved in DMF.

### Cell Counting Kit-8 (CCK-8) assay

For calculating the 50% inhibitory concentration (IC50) and evaluate cell viability, 15,000 cells were seeded in each well of a 96-well plate and treated with CDDP at different concentrations as 0 μM, 1.25 μM, 2.5 μM, 5 μM, 10 μM, 20 μM, 40 μM, and 3-MA at the concentrations as 3 mM. The absorbance at 450 nm was assessed using a microplate reader (EPOCH, BioTek Instruments, Inc.) after samples were incubated for 2 hours with 10 µL of CCK-8 reagent (GLPBIO, USA).

### Colony elimination assay

A colony forming assay was conducted to evaluate the inhibitory effects of the compounds on the clonogenic survival of NPC cells, 2 mL of DMEM complete culture medium (containing 10% FBS) with 5000 cells was added to each well of a 6-well plate. Each treatment group was allocated three replicate wells. The cells were cultured for 8 days. Subsequently, the cells were treated according to the following groups: Negative control (NC; 0.1%, v/v, DMF), 3-MA (3 mM), CDDP (20 μM), and CDDP (20 μM) + 3-MA (3 mM). After 24 hours of drug treatment, the cells were fixed for 15 minutes using 4% paraformaldehyde. Once the fixative was removed, the cells were stained at room temperature for 30 minutes with a diluted 0.1% crystal violet solution and then washed thoroughly with PBS. The formed colonies were photographed with a microscope (Lionheart, Biotek, VT, USA), and the number of colonies was analyzed using the associated Gen5 software (version 3.12). The adjusted colony forming rate was calculated with the formula: Adjusted colony forming Rate = colony forming Rate (%)/ colony forming Rate in the NC group (%).

### Immunofluorescence assay for γ-H2AX

In a 24-well plate, 1.5 × 10⁵ cells were seeded per well in 800 μL of DMEM complete medium supplemented with 10% FBS. Following cell attachment, treatments were applied according to the following groups: Negative control (NC; 0.1%, v/v, DMF), 3-MA (3 mM), CDDP (20 μM), and CDDP (20 μM) + 3-MA (3 mM). After 24h of treatment, cells were fixed, and DNA damage was assessed by immunofluorescent staining of γ-H2AX (DNA Damage Assay Kit by γ-H2AX Immunofluorescence, Beyotime Institute of Biotechnology). DAPI was used to stain the nucleus and captured under microscope (Lionheart, Biotek, VT, USA).

### Cell cycle detection

In a 24-well plate, 1.5 × 10⁵ cells were seeded per well in 800 μL of DMEM complete medium supplemented with 10% FBS. Following cell attachment, treatments were applied according to the following groups: Negative control (NC; 0.1%, v/v, DMF), 3-MA (3 mM), CDDP (20 μM), and CDDP (20 μM) + 3-MA (3 mM). After 24 h of treatment, cells were collected and fixed in 75% alcohol. After another 24-hour period at 4˚C, the 75% alcohol was removed through centrifugation at 650 x g for 5 minutes at 4˚C. Subsequently, the cells were washed twice with cold PBS. For staining, the cells were treated with propidium iodide and RNase. They were then incubated in the dark at 37˚C for 20 minutes. The final concentration of the PI solution (from Beyotime Institute of Biotechnology) was set at 50 µg/mL, and the RNase solution (also from Beyotime Institute of Biotechnology) was at a final concentration of 100 µg/mL. The cell cycle distribution was analyzed by measuring the DNA content using a Flow cytometer (NovoCyte, Agilent, United States). The acquired data were then processed and analyzed with NovoExpress software (Agilent, United States).

### JC-1 assay

In a 12-well plate, 3 × 10⁵ cells were seeded per well in 1 mL of DMEM complete medium supplemented with 10% FBS. Following cell attachment, treatments were applied according to the following groups: Negative control (NC; 0.1%, v/v, DMF), 3-MA (3 mM), CDDP (20 μM), and CDDP (20 μM) + 3-MA (3 mM). After 24 h of treatment, mitochondrial membrane potential was analyzed using the JC-1 Mitochondrial Membrane Potential Assay Kit (Beyotime Institute of Biotechnology), which detects early apoptotic events through fluorescence shifts indicative of mitochondrial depolarization. The proportion of mitochondrial membrane potential changed cells was measured using the Flow cytometer (NovoCyte, Agilent, United States), and the data were analyzed using NovoExpress software (Agilent Technologies).

### Cell apoptosis detection

In a 12-well plate, 3 × 10⁵ cells were seeded per well in 1 mL of DMEM complete medium supplemented with 10% FBS. Following cell attachment, treatments were applied according to the following groups: Negative control (NC; 0.1% DMF, v/v), 3-MA (3 mM), CDDP (20 μM), or a combination of CDDP (20 μM) and 3-MA (3 mM). After 24 h of treatment, cell apoptosis was assessed using the Annexin V-FITC/PI Apoptosis Detection Kit (Dalian Meilun Biotechnology, Dalian, China). Briefly, cells were stained with 5 μL of Annexin V-FITC (40 µg/mL) and 10 μL of PI (50 µg/mL), incubated for 15 minutes at room temperature in the dark, and then resuspended in 400 μL of 1X Binding Buffer. Apoptotic cell populations were quantified using a NovoCyte flow cytometer (NovoCyte, Agilent, United States), and the data were analyzed with NovoExpress software (Agilent Technologies, United States). Apoptotic cells were classified as either early apoptotic (Annexin V-FITC positive/PI negative) or late apoptotic (Annexin V-FITC positive/PI positive).

### Western blotting

In each well of a 12-well plate, 800 μL of DMEM complete culture medium (contain-ing 10% FBS) with 3 x10^5^ cells was added in preparation for subsequent experiment. After cell adhesion, the cells were assigned to one of the following treatment groups: Negative control (NC; 0.1% DMF, v/v), 3-MA (3 mM), CDDP (20 μM), or a combination of CDDP (20 μM) and 3-MA (3 mM). Following treatment, the cells were lysed using RIPA (Strong) Buffer (Cwbiotech) to extract total protein. Protein concentrations were quantified using the BCA Protein Assay Kit (Beyotime Institute of Biotechnology) following the manufacturer’s protocol. Equal amounts of protein samples were then separated via SDS-PAGE and subsequently transferred onto PVDF membranes (MilliporeSigma) for further analysis. After membrane transfer, blocking was performed with 5% non-fat milk in PBST (0.05% Tween-20) for 1 hour at room temperature. The membrane was then washed twice with PBST and incubated overnight at 4°C with the primary antibody. After three 10-minute PBST washes, the membrane was incubated with the secondary antibody for 1 hour at room temperature, followed by three 5-minute PBST washes. The HRP-labeled protein-antibody complexes were identified by chemiluminescence imaging using Super ECL reagent (Dalian Meilun Biotechnology, China) and imaged with an Azure C280 imager (Azure Biosystems, USA). GAPDH (1:5000, EM1101, HuaAnBio, Hangzhou, China) was used as a control. Caspase-9 antibody (1:1000, 9502), Caspase-3 antibody (1:1000, 9662) and poly (ADP-ribose) polymerase (PARP) antibody (1:1000, 9532) were purchased from Cell Signaling Technology, Inc (Danvers, Massachusetts, USA). Bcl-2 antibody (1:2000, ET1603-11) and Bax antibody (1:2000, ET1603-34) were purchased from Huaan Biotechnology (Hangzhou, China). γ-H2AX (1:1000, ab81299, Abcam, Cambridge, UK). P53 antibody (1:1000, ab81299, Abcam, Cambridge, UK). ATM antibody (1:1000, A18257), Phospho-ATM (S1981) antibody (1:1000, AP1030), ATR antibody (1:1000, A21253) and Phospho-ATR (S428) antibody (1:1000, AP1358) were purchased from ABclonal (Wuhan, China). P53 antibody (1:5000, 60283, ProteinTech, Wuhan, China), Phospho-p53 (S15) antibody (1:1000, HA721756, Huaan Biotechnology, Hangzhou, China), Phospho-p53 (S46) antibody (1:1000, ab76242, Abcam, Cambridge, UK). Anti-rabbit IgG, HRP-linked antibody (1:2000, 7074) and anti-mouse IgG, HRP-linked antibody (1:2000, 7076) were purchased from Cell Signaling Technology, Inc (Danvers, Massachusetts, USA). Pageruler #26616 and #26619 (ThermoFisher, Waltham, MA, USA) was used as the molecular marker. Protein bands were semi-quantified using ImageJ (v1.42q; NIH, Bethesda, MD, USA). Grayscale values were normalized against GAPDH, and relative protein levels were compared across groups.

### Statistical analysis and mapping

All experiments were performed in triplicate, and data are presented as mean ± standard deviation (SD). Statistical analyses were conducted using nonparametric methods: the Mann-Whitney U test was used for comparisons between two groups, and the Kruskal-Wallis test followed by Bonferroni correction was employed for multiple group comparisons. GraphPad Prism version 8.3.0 (GraphPad Software, Inc.) was used for data visualization, and statistical analyses were performed using SPSS Statistics version 25 (IBM Corp., Armonk, NY, USA). Statistical significance was defined as *P* < 0.05. The schematic illustration was drawn using Figdraw tools (Hangzhou Duotai Technology Co., Ltd., Hangzhou, China).

## Results

### 3-MA enhances the inhibitory effect of CDDP on the cell viability of NPC cells

**Fig 1:**
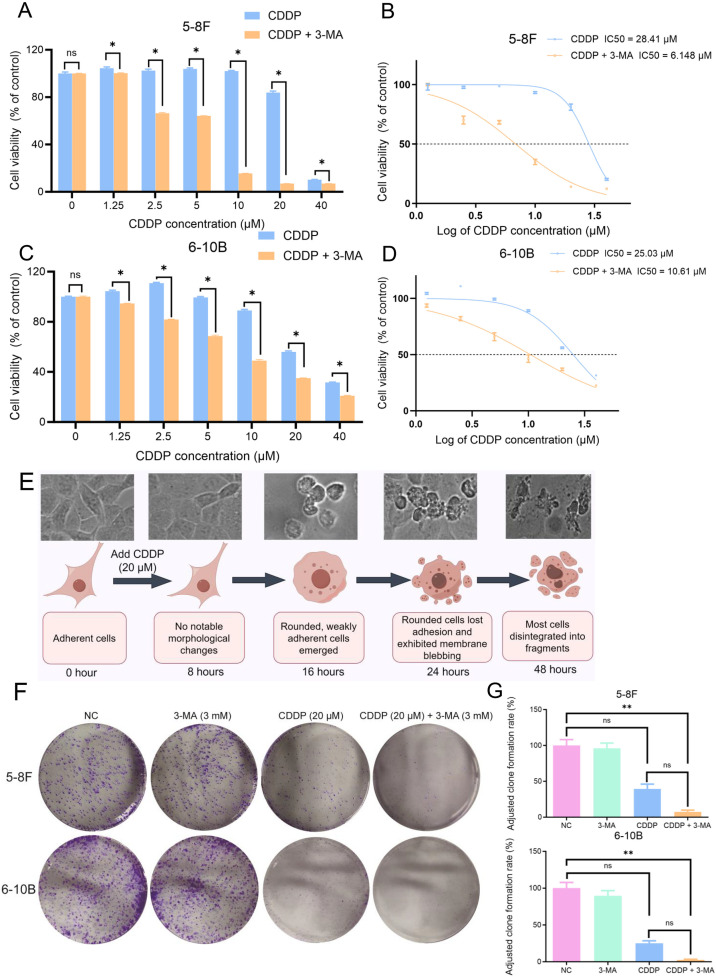
Enhancement of CDDP-induced proliferation inhibition in nasopharyngeal cancer cells by 3-MA. **(A, C)** CCK8 assay results showed that after 24 hours of treatment with a combination of 3 mM 3-MA and various concentrations of CDDP, the cell viability of nasopharyngeal cancer cells was lower than that of the CDDP-only treatment group. The data are presented as the mean ± SD (n = 3), and the Mann-Whitney U test was used for comparisons between two groups. * present vs. CDDP (20 μM) group at 24h, *P < 0.05, *ns* means not significant. **(B, D)** The combination of 3-MA and CDDP reduced the IC50 of CDDP against 5-8F and 6-10B nasopharyngeal cancer cells within 24 hours. **(E)** Schematic timeline of experimental design. **(F)** Colony elimination assay results indicated that the combination of 3-MA (3 mM) and CDDP (20 μM) significantly reduced the clonogenic survival rate of nasopharyngeal cancer cells compared to CDDP (20 μM) treatment alone. The data are presented as the mean ± SD (n = 3) and Kruskal-Wallis test followed by Bonferroni correction was employed for multiple group comparisons. * present vs. CDDP (20 μM), *P < 0.05, **P < 0.01, *ns* means not significant.

The CCK-8 assay was used to assess cell viability and determine the 50% inhibitory concentration (IC50). After 24 hours of drug treatment in 5-8F cells, starting at a CDDP concentration of 2.5 μM, cell viability in the 3-MA plus CDDP group was significantly lower than in the CDDP-only group. This combination treatment reduced the IC50 of CDDP from 28.41 μM to 6.148 μM (Fig 1A, 1B). Similarly, in 6-10B cells treated for 24 hours, starting at a CDDP concentration of 1.25 μM, cell viability was markedly reduced in the 3-MA plus CDDP group compared to the CDDP-only group. The combined treatment lowered the IC50 of CDDP from 25.03 μM to 10.61 μM (Fig 1C, 1D). Cell proliferation changes following 48-hour drug treatment were assessed using the CCK-8 assay (S1 Fig). Based on the observed results, a cisplatin concentration of 20 μM and a treatment duration of less than 24 hours were selected for subsequent experiments. This optimization was aimed at improving experimental efficiency while avoiding confounding effects caused by excessive differences between groups at 48 hours, such as nutrient deprivation-induced cell death in the control group or excessive cell loss in the 3-MA plus CDDP group that may hinder detection of internal reference proteins.

A schematic timeline was constructed to illustrate the experimental design, highlighting the key time points from 0 to 48 hours after drug treatment initiation (Fig 1E). Consistent with these findings, the colony formation assay further demonstrated that NPC cells treated with both CDDP and 3-MA exhibited the lowest colony formation rate, which was significantly lower than that observed in the NC group (Fig 1F, 1G).

### 3-MA enhanced SubG1 phase distribution in CDDP-treated NPC cells

**Fig 2:**
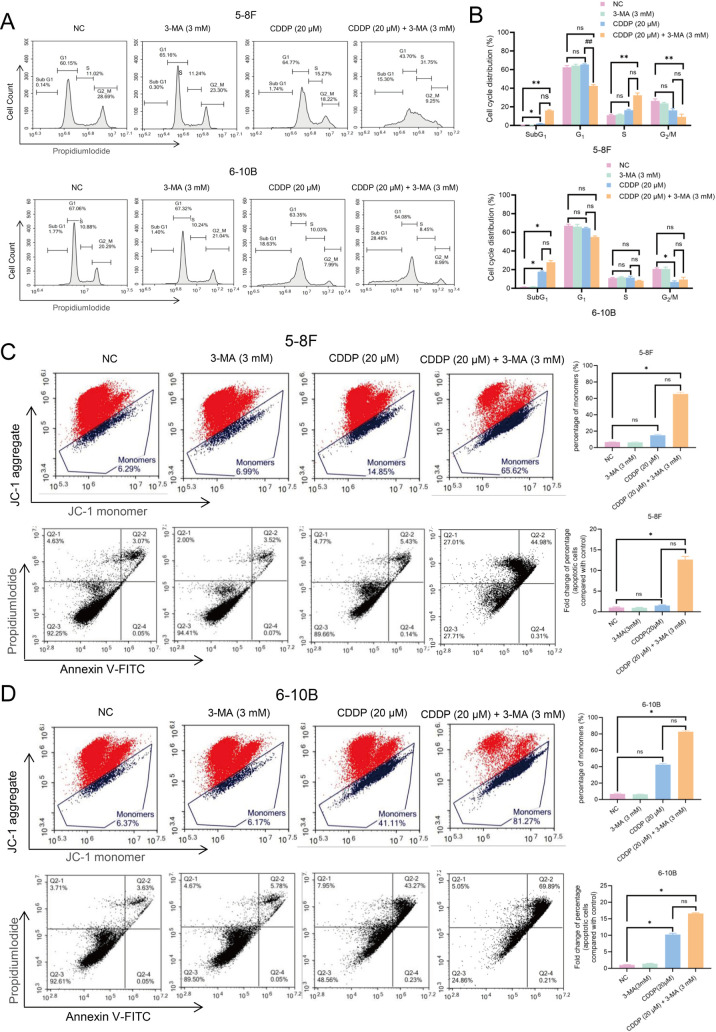
The combination of 3-MA and CDDP significantly increased CDDP-induced cell death. **(A, B)** The distribution of the Sub-G1 phase in nasopharyngeal cancer cells treated with 3-MA and CDDP for 24 hours was significantly higher compared to both the CDDP-only treatment group and the control group. The data are presented as the mean ± SD (n = 3) and Kruskal-Wallis test followed by Bonferroni correction was employed for multiple group comparisons. * present vs. NC group, # present vs. CDDP (20 μM), *P < 0.05, **P < 0.01, *ns* means not significant. **(C, D)** After 24 hours of treatment with 3-MA and CDDP, nasopharyngeal cancer cells showed a significant increase in JC-1 monomer fluorescence compared to both the CDDP-only treatment group and the control group (C, D, top rows). Additionally, the proportion of dead cells was also significantly higher (C, D, bottom rows). The data are presented as the mean ± SD (n = 3) and Kruskal-Wallis test followed by Bonferroni correction was employed for multiple group comparisons. * present vs. NC group, # present vs. CDDP (20 μM), *P < 0.05, **P < 0.01, *ns* means not significant.

To evaluate the impact of 3-MA in combination with CDDP on cell cycle distribution and cell death in nasopharyngeal carcinoma cells, DNA content analysis was performed using flow cytometry following 24 hours of drug treatment. Both CDDP alone and the combination of 3-MA with CDDP significantly increased the accumulation of NPC cells in the SubG1 phase. Additionally, both treatment conditions induced S phase arrest in 5-8F cells; however, no significant S phase arrest was observed in 6-10B cells. Since the increase of SubG1 phase in 6-10B cells after CDDP treatment is higher than that in 5-8F cells, it is speculated that this proportion may be caused by cell death blocked in S phase (Fig 2A, 2B). These findings from the cell cycle analysis presented that while 3-MA does not induce significant change of cell cycle distribution in NPC cells, and it does promote SubG1 phase accumulated, suggested that more cell death in 3-MA combined with CDDP treated NPC cells than CDDP treated alone cells.

### 3-MA potentiates CDDP-induced DNA damage related cell death in NPC cells

Apoptosis is a key outcome of DNA damage-induced cell death. Early apoptotic cells are characterized by mitochondrial membrane potential disruption, which causes JC-1 aggregates to convert into monomers, indicating mitochondrial depolarization. The combination of 20 μM CDDP with 3 mM 3-MA significantly increased the proportion of JC-1 monomers in NPC cells compared to the control group. A similar increasing trend was observed in the CDDP-only group; however, the difference was not statistically significant. Although the combination treatment induced a higher proportion of JC-1 monomers compared to CDDP alone, this increase also did not reach statistical significance (Fig 2C, 2D, top rows). Annexin V-FITC/PI staining further confirmed this trend, showing an increase trend in apoptotic cell percentage in both the CDDP-only and combination treatment groups compared to the control. Moreover, cells treated with both CDDP and 3-MA exhibited a significantly higher apoptotic rate than NC groups. Collectively, these findings demonstrate that 3-MA effectively enhances CDDP-induced apoptosis in NPC cells (Fig 2C, 2D, bottom rows).

### 3-MA enhanced CDDP-induced apoptotic protein expression in NPC cells

**Fig 3:**
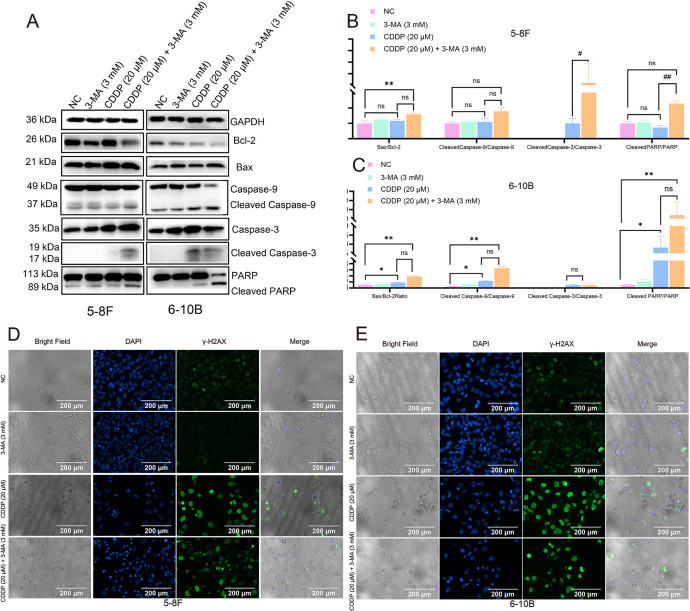
The combination of 3-MA and cisplatin enhances the initiation of apoptotic signaling and induces DNA damage in nasopharyngeal cancer cells. (A-C) After 24 hours of treatment with 3-MA and CDDP, nasopharyngeal cancer cells exhibited significantly increased ratio of Bax/Bcl2, cleaved caspase-9/full caspase-9, cleaved caspase-3/full caspase-3, and cleaved-PARP/full PARP compared to both the CDDP-only treatment group and the control group. The data are presented as the mean ± SD (n = 3) and Kruskal-Wallis test followed by Bonferroni correction was employed for multiple group comparisons. * present vs. NC group, # present vs. CDDP (20 μM), *P < 0.05, **P < 0.01, *ns* means not significant. (D, E) Immunostaining results demonstrated that after 16 hours of treatment with CDDP, Compared to the 3-MA alone and control groups, cells treated with CDDP alone or in combination with 3-MA exhibited stronger γ-H2AX (green) fluorescence signals and a greater number of morphologically abnormal nuclei (blue). Scale bar = 200 μm.

We examined apoptotic protein expression 24 hours post-treatment, aligning with the observed drug-induced cell death timeline. In 5-8F cells, the combination of CDDP and 3-MA downregulated Bcl-2 expression and significantly elevated the Bax/Bcl-2 ratio compared to NC group, indicating a reduction in anti-apoptotic capacity [16]. This effect was accompanied by elevated levels of cleaved forms of caspase-9, caspase-3, and PARP, along with a higher cleaved-to-total ratio for these proteins, confirming the activation of apoptotic signaling pathways [17]. In 6-10B cells, both CDDP alone and the combination treatment increased the Bax/Bcl-2 ratio and enhanced cleaved caspase-9, caspase-3, and PARP levels (Fig 3A–3C). Notably, the combination treatment resulted in a more pronounced upregulation of these apoptotic markers, and further supporting the enhanced apoptotic response.

### 3-MA accelerated the termination of DNA damage repair process in NPC cells

γ-H2AX serves as a key marker of DNA damage and repair signaling [18]. Immunofluorescence analysis at 16 hours revealed that γ-H2AX expression was significantly increased in cells treated with cisplatin (CDDP), both alone and in combination with 3-Methyladenine (3-MA). Notably, 5-8F and 6-10B cells in the CDDP + 3-MA group exhibited a greater number of condensed or collapsed nuclei compared to the CDDP-alone group. In some of these condensed nuclei, γ-H2AX signal intensity was reduced. Due to the higher sensitivity of 6-10B cells to CDDP compared to 5-8F cells, a larger proportion of 6-10B cells lost adhesion and underwent fragmentation. As a result, immunofluorescence staining could only capture the nuclear morphology and γ-H2AX signal in the remaining adherent 6-10B cells. And this detachment became more pronounced at 24 hours post-treatment in 6-10B cells (S2 Fig). Importantly, these detached cells were collected during flow cytometry assays to assess apoptosis. Overall, the γ-H2AX and DAPI immunofluorescence findings were consistent with the apoptotic patterns observed in these flow cytometry detections and western blot analysis (Fig 3D, 3E).

**Fig 4:**
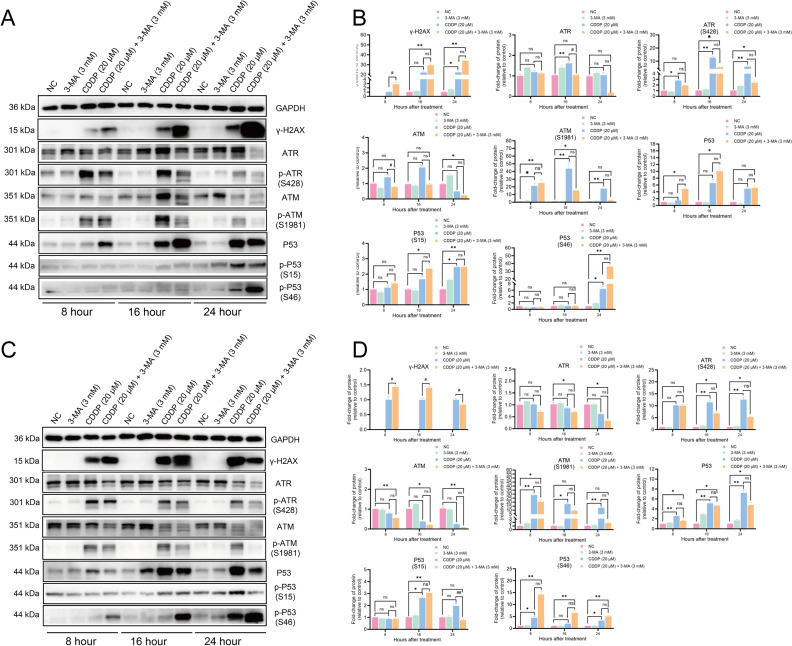
3-MA accelerates the termination of DNA damage repair and activates p53-mediated apoptosis in nasopharyngeal carcinoma cells. **(A, C)** Expression levels and quantification of DNA damage repair-related proteins in 5-8F NPC cells following drug treatment for 8, 16, and 24 hours. **(D, E)** Expression levels and quantification of DNA damage repair-related proteins in 6-10B NPC cells following drug treatment for 8, 16, and 24 hours. Representative western blot results were analyzed using Kruskal-Wallis test followed by Bonferroni correction for multiple group comparisons. * present vs. NC group, # present vs. CDDP (20 μM), *P < 0.05, **P < 0.01, *ns* means not significant.

We further explored the ATR/ATM/p53 pathway, which mediates DNA damage response. CDDP alone stimulated this pathway, increasing ATR, ATM, and p53 levels, indicating active repair. However, 3-MA accelerated the termination of this response. Western blot results showed that the combined treatment led to a premature decrease in ATR, ATM, and total p53. Moreover, the observed shift in p53 phosphorylation, with reduced Ser15 and increased Ser46 phosphorylation, suggested a switch from DNA repair maintenance to apoptosis induction (Fig 4A–4D).

## Discussion

Targeting the DNA damage response (DDR) pathway to selectively eliminate tumor cells with genomic instability represents a fundamental and pivotal strategy in cancer therapy. In recent years, this approach has regained attention due to the successful application of DNA damage synthetic lethality-based therapeutics [19]. In 2014, olaparib, the first cancer drug based on this strategy, was approved for the treatment of ovarian cancer and has since become a first-line therapy [20,21]. The clinical success of PARP inhibitors has strongly validated the application of the “synthetic lethality” concept in drug development under DNA damage conditions, establishing it as a key direction for future cancer therapies [22,23].

In 2024, multiple clinical trials explored DNA repair-targeted synthetic lethality drugs. SIM0501 (USP1 inhibitor) disrupted DDR-associated ubiquitination to suppress tumor proliferation [24]; GH2616 inhibited KIF18A, impairing spindle-centromere attachment [25]; AZD3470, a second-generation PRMT5 inhibitor, selectively targeted MTA-deficient tumors with reduced toxicity [26]; and HRO761 promoted WRN degradation, activating p53-mediated apoptosis in microsatellite instability tumors [27].

**Fig 5:**
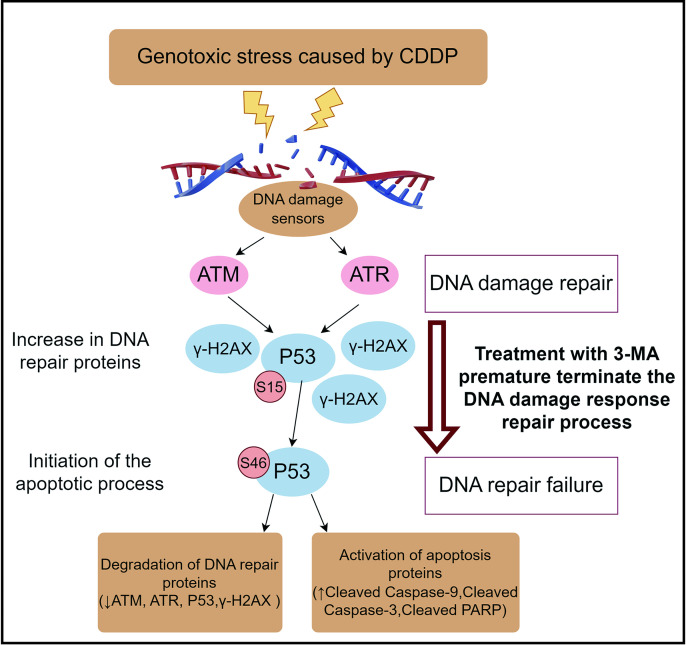
Schematic representation of 3-MA-induced premature termination of DNA damage repair and activation of apoptotic signaling.

In this study, we utilized cisplatin (CDDP) to actively induce DNA damage and observed that the addition of 3-MA not only enhanced the inhibitory effect of CDDP on the proliferation of nasopharyngeal carcinoma (NPC) cells but also resulted in a higher rate of cell death within 24 hours. By disrupting cell cycle progression, reducing mitochondrial membrane potential (JC-1 staining), and promoting apoptosis, 3-MA was shown through flow cytometry to enhance CDDP-induced cell death in NPC cells. Furthermore, western blot analysis of protein profiles suggested that 3-MA accelerates the termination of the DNA damage repair process induced by CDDP and initiates apoptotic signaling (Fig 5).

In several tumor-related studies, 3-MA has also been observed to enhance the effects of cell cycle-targeting agents; however, mechanistic investigations have largely stagnated [28–30]. Most studies either focus solely on changes in apoptotic proteins or on autophagy, which may not be a representative cellular process. It is important to note that autophagy fluctuates throughout the cell cycle and is significantly suppressed during mitosis [31]. Moreover, 3-MA has been reported to exert bidirectional effects on autophagy, acting as both an inhibitor and an inducer under different contexts [13]. Our findings offer a novel perspective for further exploration of the interaction between 3-MA and cell cycle-targeting therapies. We suggest that combining CDDP with 3-MA holds promise as a potential chemotherapy strategy for enhancing DNA damage-induced cell death. Given 3-MA’s low toxicity and its ability to significantly lower the IC50 of CDDP, this combination may allow for reduced cisplatin dosage, potentially minimizing adverse effects and resistance. Furthermore, this approach could be extended to other therapeutic strategies targeting DNA damage pathways.

However, the precise signaling pathways through which 3-MA induces premature termination of the DNA damage repair process remain unclear. As a byproduct of DNA alkylation damage, 3-MA is recognized and excised by N-methylpurine DNA glycosylase (MPG), a key enzyme in the base excision repair (BER) pathway, suggesting a potential mechanistic link between 3-MA and DDR regulation. Based on this, we hypothesized that 3-MA may potentiate the effects of cisplatin by interfering with DDR pathways. 3-MA may competitively disrupt the interaction between MPG and p53 [32]. Additionally, high intracellular concentrations of 3-MA may signal extensive DNA damage, implying a potential role for 3-MA levels in modulating the cellular threshold for DNA damage recognition. Moving forward, we will continue to investigate these underlying mechanisms and provide valuable theoretical insights into the mechanisms of DNA damage repair.

Although early-stage cancer patients have benefited from improvements in diagnosis, surgical interventions, and long-term monitoring, treatment outcomes for advanced-stage malignancies remain suboptimal and continue to pose major clinical challenges [33]. Cisplatin is widely used in the treatment of advanced nasopharyngeal carcinoma; however, the emergence of drug resistance limits its long-term efficacy. The high selection pressure imposed by chemotherapy may facilitate the clonal evolution of more aggressive and treatment-resistant tumor cells, potentially leading to hyperprogression. This highlights the complexity of therapeutic decision-making in advanced cancer, where the balance between curative intent and palliative care must be carefully evaluated [34,35]. In this context, strategies that enhance drug sensitivity and reduce effective dosing may help mitigate toxicity while maintaining therapeutic benefit. Nevertheless, the translation of preclinical findings into clinical practice remains a substantial hurdle. Bridging this gap will require not only further mechanistic insight but also rigorous validation through well-designed clinical trials.

## Conclusions

Our study demonstrated that NPC cells treated with a combination of CDDP and 3-MA exhibited increased cell death. We propose that 3-MA accelerates the termination of the DNA damage repair process triggered by CDDP, thereby activating apoptotic signaling pathways. These findings suggest that 3-MA may function as a potential sensitizer to enhance the efficacy of CDDP in NPC chemotherapy.

## Supporting information

S1 Fig Enhancement of CDDP-induced proliferation inhibition in nasopharyngeal cancer cells by 3-MA at 48 hours. (A, C) CCK8 assay results showed that after 48 hours of treatment with a combination of 3 mM 3-MA and various concentrations of CDDP, the cell viability of nasopharyngeal cancer cells was lower than that of the CDDP-only treatment group. The data are presented as the mean ± SD (n = 3), and the Mann-Whitney U test was used for comparisons between two groups. * present vs. CDDP (20 μM) group at 48h, *P < 0.05, *ns* means not significant. (B, D) The combination of 3-MA and CDDP reduced the IC50 of CDDP against 5-8F and 6-10B nasopharyngeal cancer cells at 48 hours.
(TIF)

S2 Fig Immunofluorescence results at 24 hours post-treatment. (A) 5-8F cells. (B) 6-10B cells. Compared to the 3-MA alone and control groups, cells treated with CDDP alone or in combination with 3-MA exhibited stronger γ-H2AX (green) fluorescence signals and a greater number of morphologically abnormal nuclei (blue). Scale bar = 200 μm.
(TIF)

S3 File Raw images of western blot experiments. (PDF)

S4 File STR analysis report for 5-8F cell line. (PDF)

S5 File STR analysis report for 6-10B cell line. (PDF)

S6 File Minimal data set. (XLSX)
