# Supplementary figures and images for: Premature termination of DNA Damage Repair by 3-Methyladenine potentiates cisplatin cytotoxicity in nasopharyngeal carcinoma cells
Source: PLoS One. 2025 Aug 4;20(8):e0329272. doi: 10.1371/journal.pone.0329272 (PMC12321125; doi:10.1371/journal.pone.0329272)

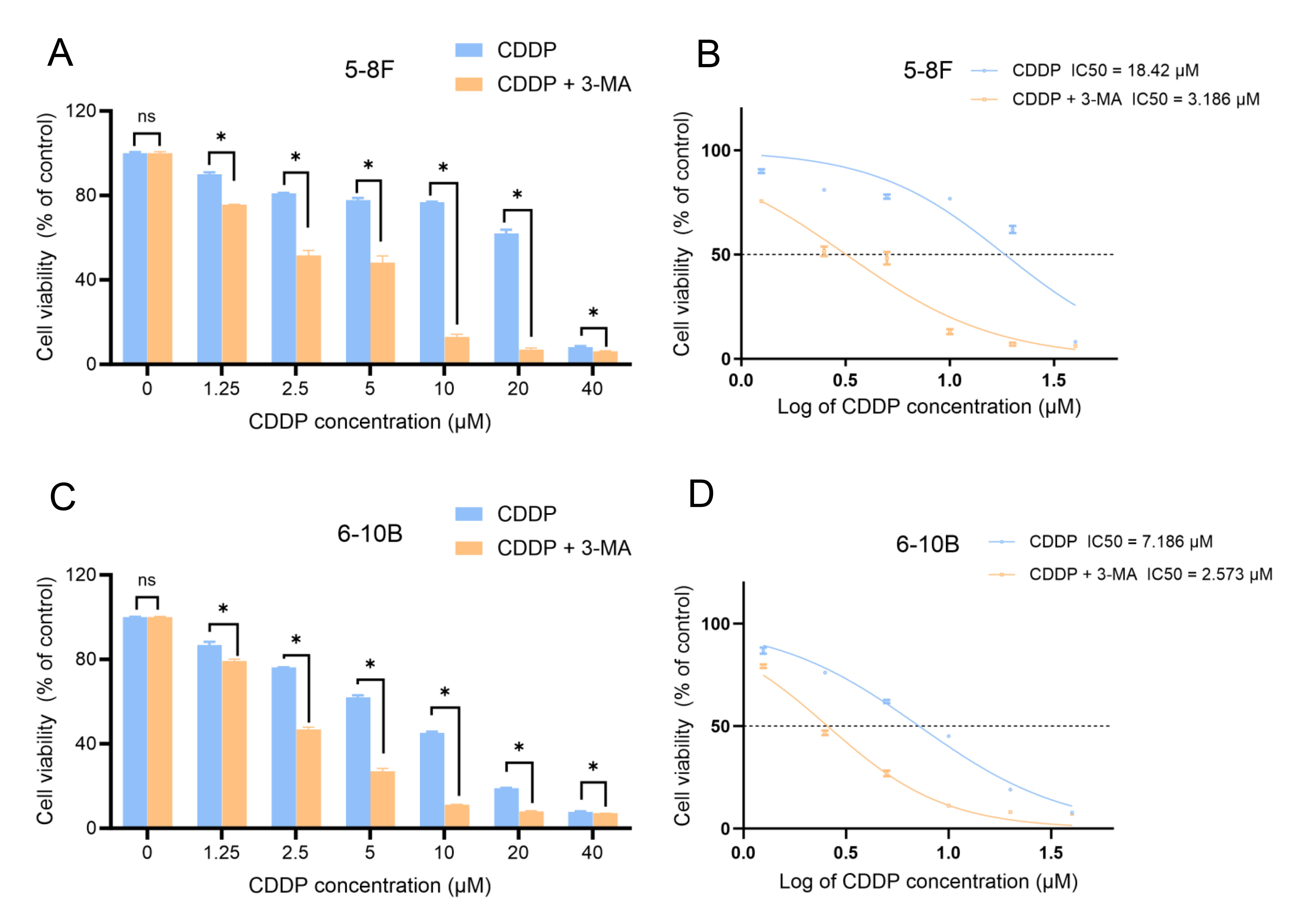

Supplement: S1 Fig — (A, C) CCK8 assay results showed that after 48 hours of treatment with a combination of 3 mM 3-MA and various concentrations of CDDP, the cell viability of nasopharyngeal cancer cells was lower than that of the CDDP-only treatment group. The data are presented as the mean ± SD (n = 3), and the Mann-Whitney U test was used for comparisons between two groups. * present vs. CDDP (20 μM) group at 48h, *P < 0.05, ns means not significant. (B, D) The combination of 3-MA and CDDP reduced the IC50 of CDDP against 5-8F and 6-10B nasopharyngeal cancer cells at 48 hours. (TIF) [file pone.0329272.s001.tif]

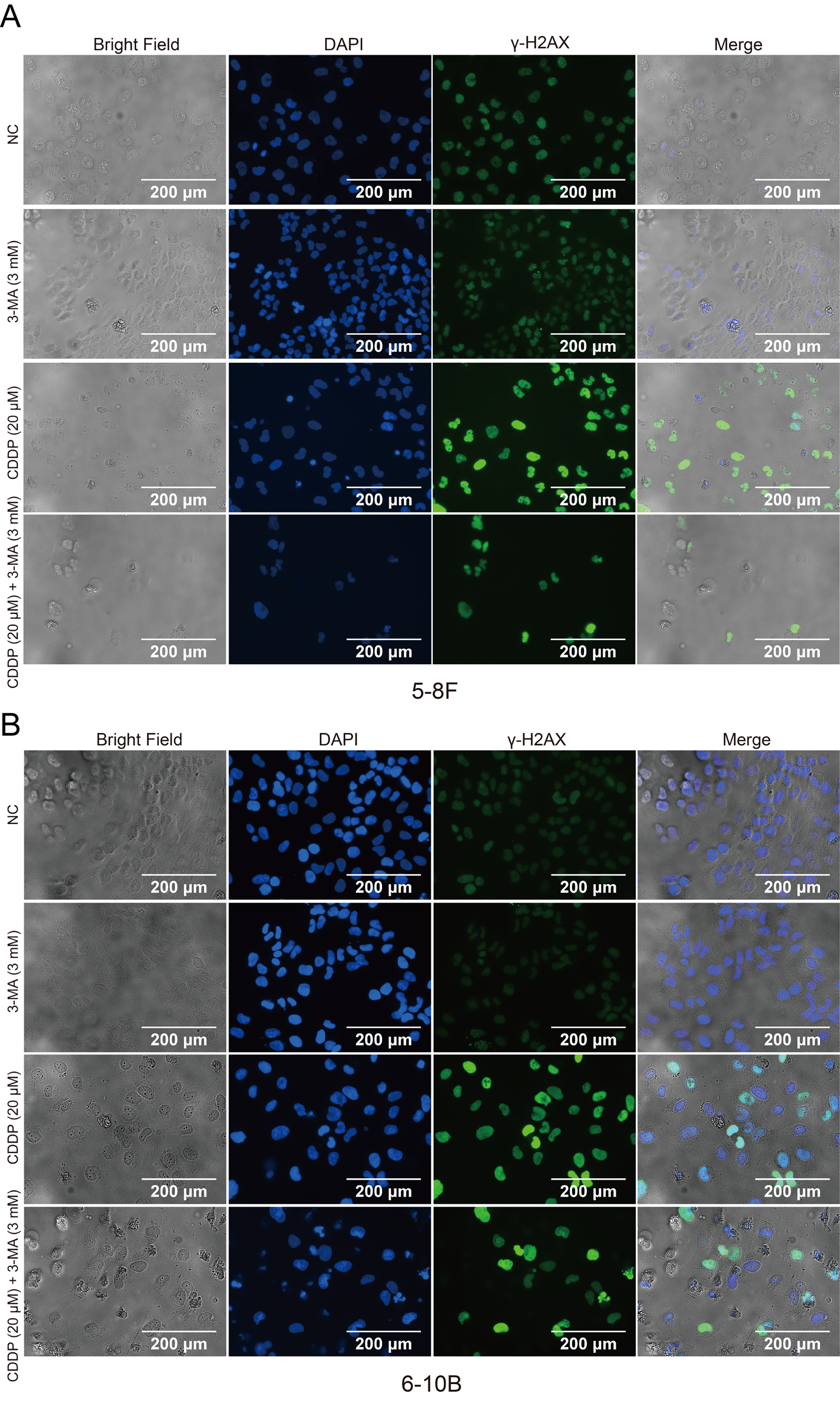

Supplement: S2 Fig — (A) 5-8F cells. (B) 6-10B cells. Compared to the 3-MA alone and control groups, cells treated with CDDP alone or in combination with 3-MA exhibited stronger γ-H2AX (green) fluorescence signals and a greater number of morphologically abnormal nuclei (blue). Scale bar = 200 μm. (TIF) [file pone.0329272.s002.tif]

Figure 3A

5-8F:

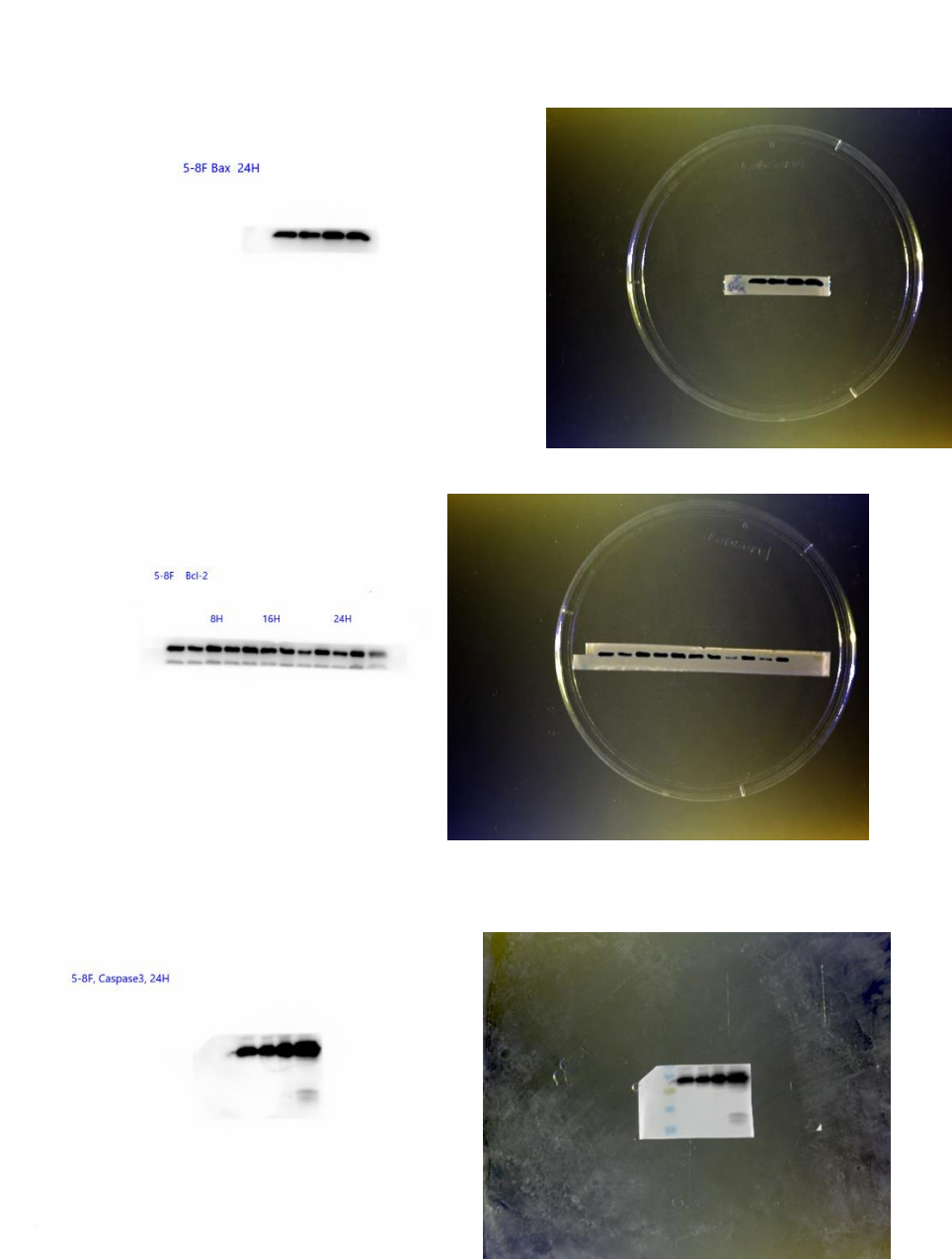

5-8F, Caspase 9, 24H, repeats

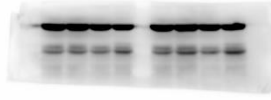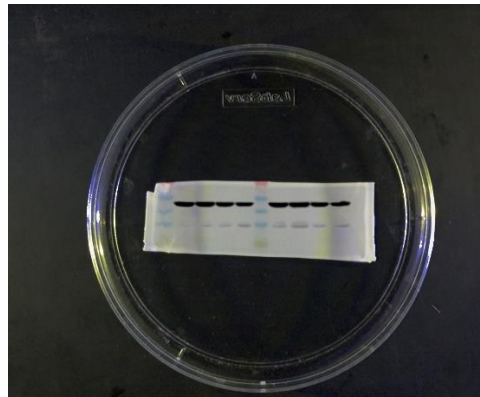

PARP, 12H, 24H

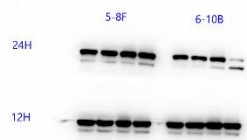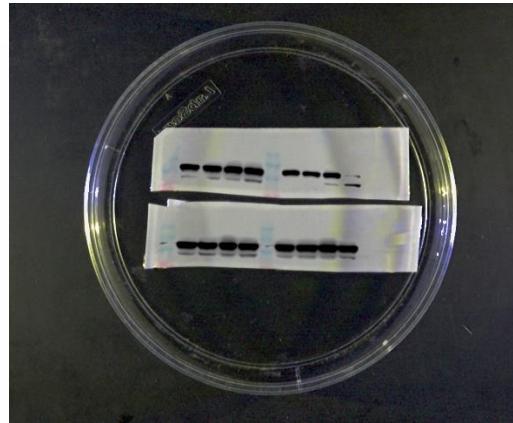

24H

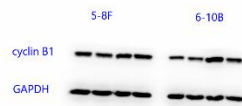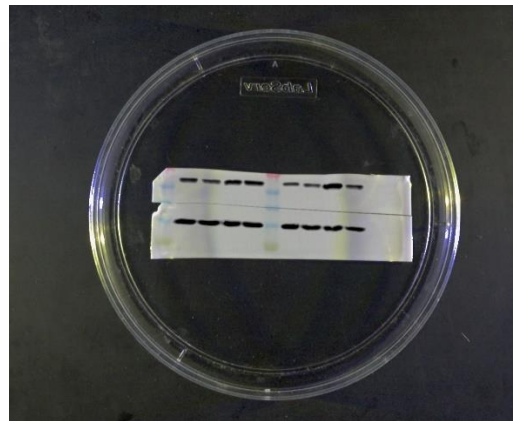

6-10B:

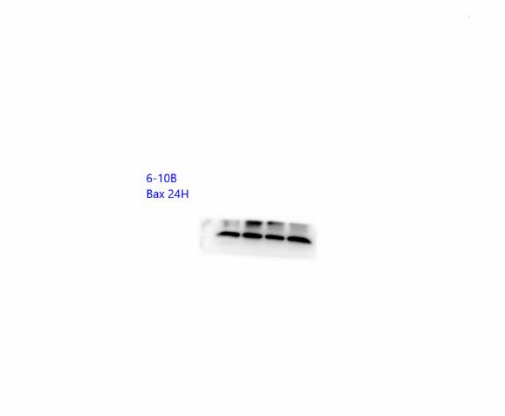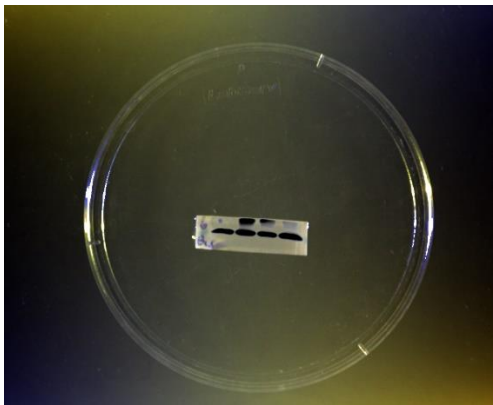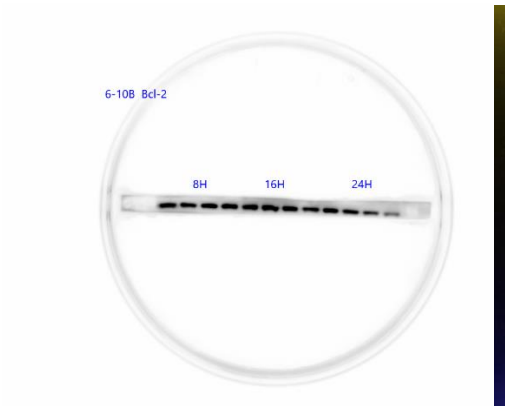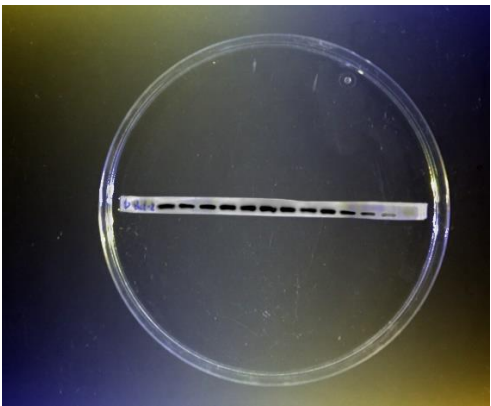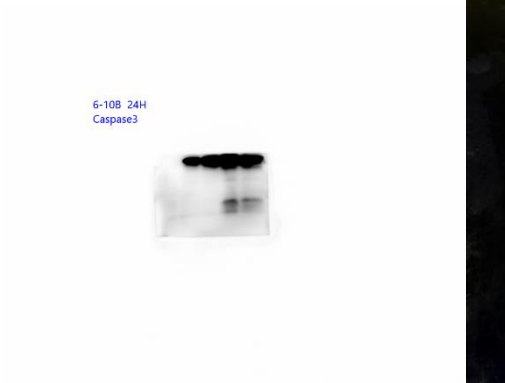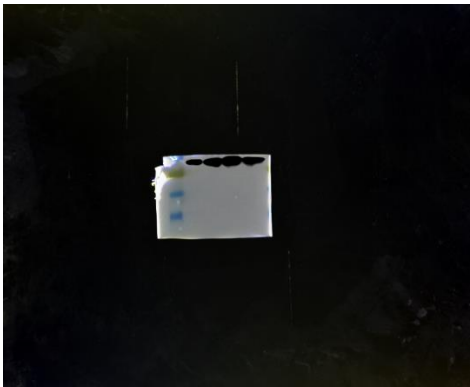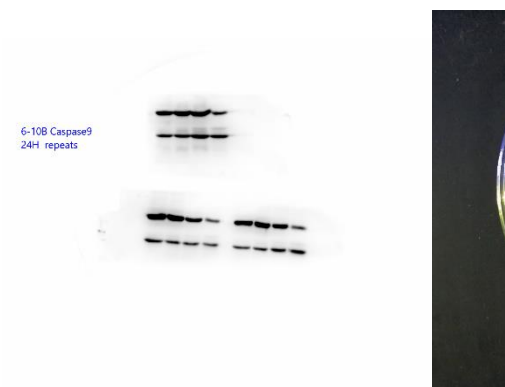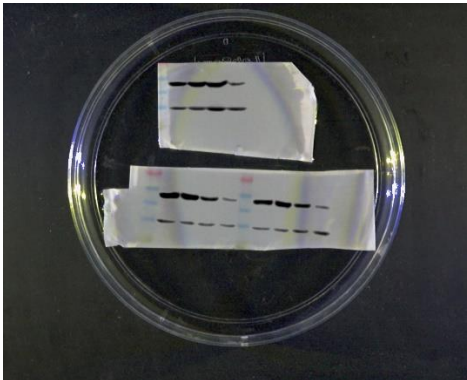

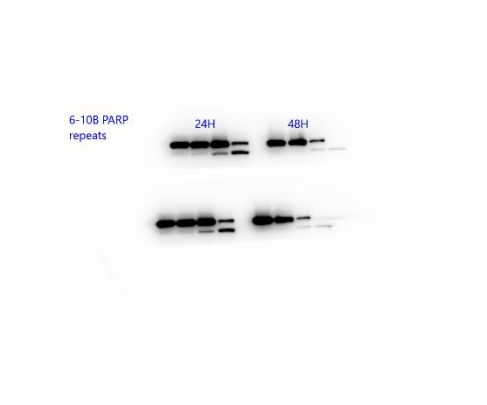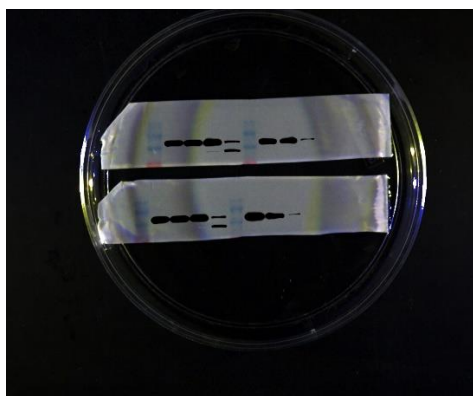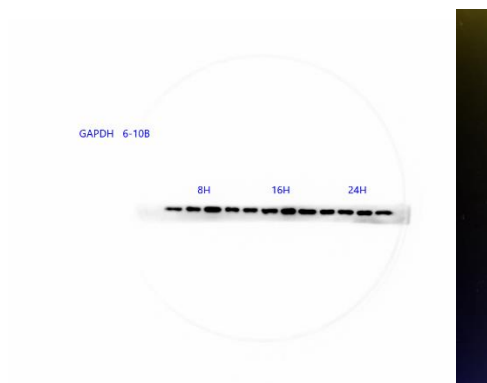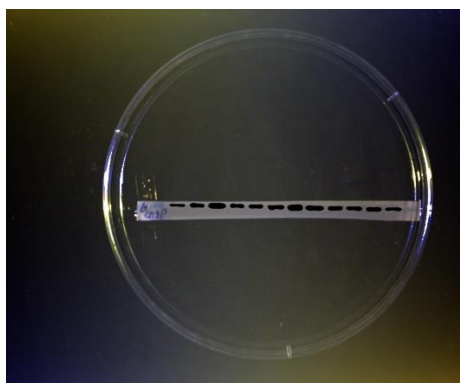

Figure 4

5-8F:

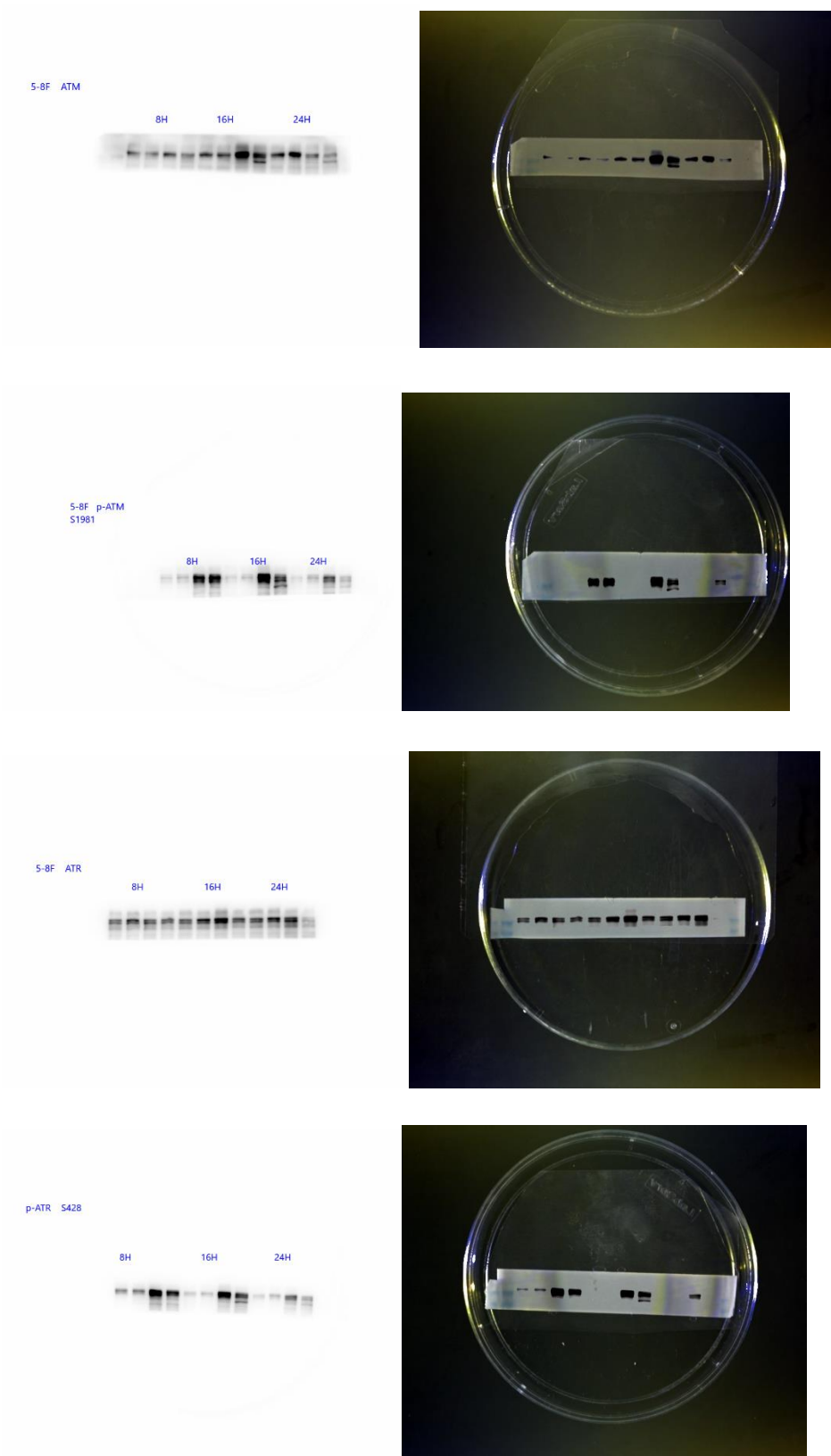

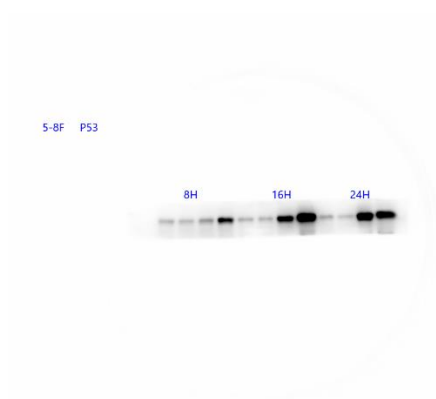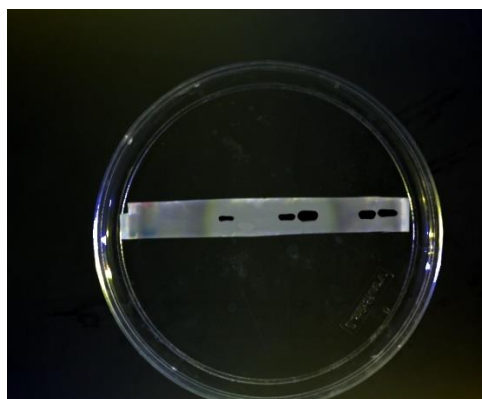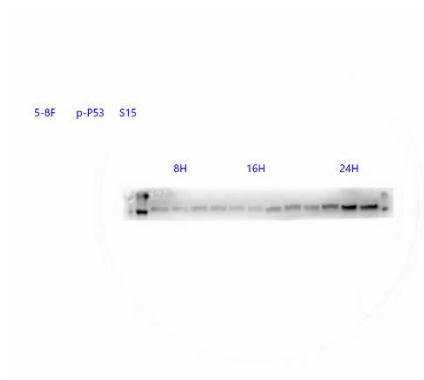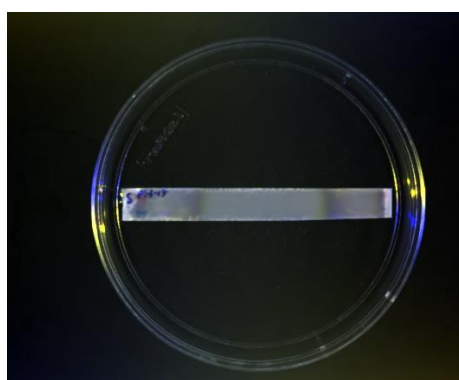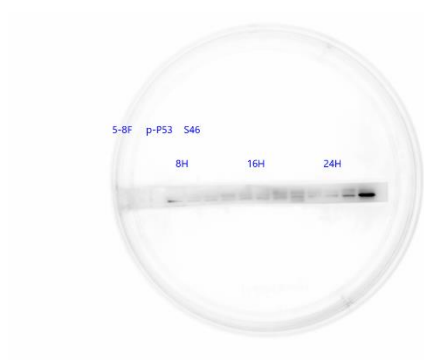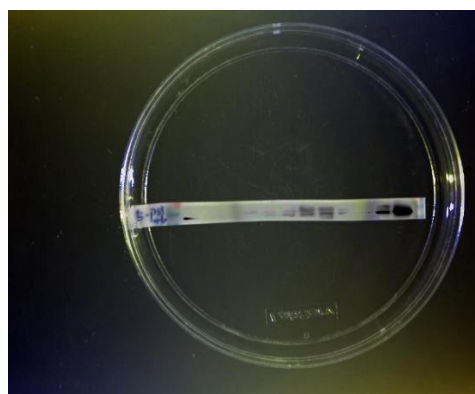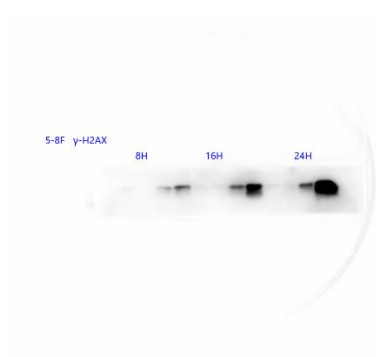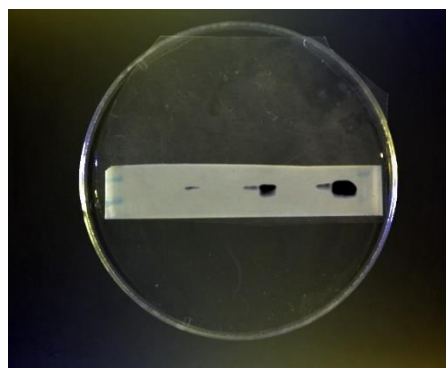

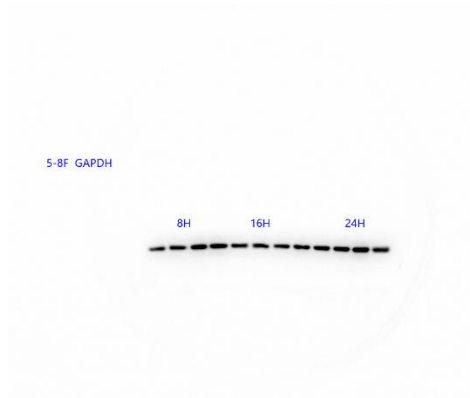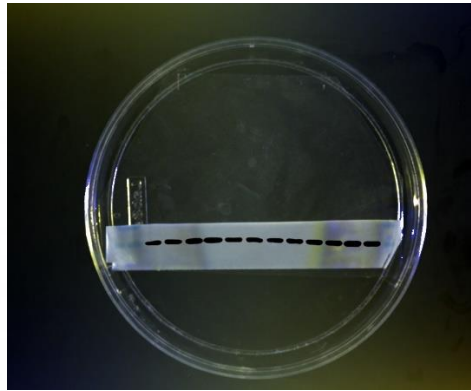

6-10B:

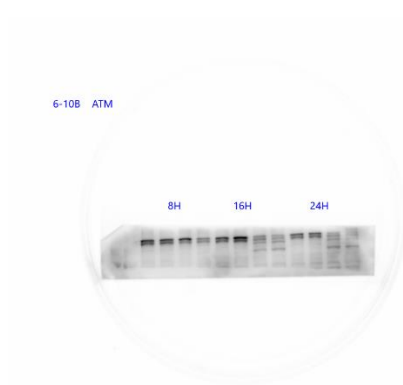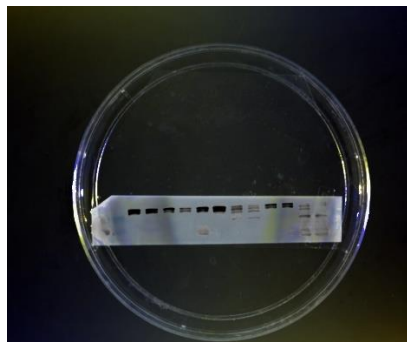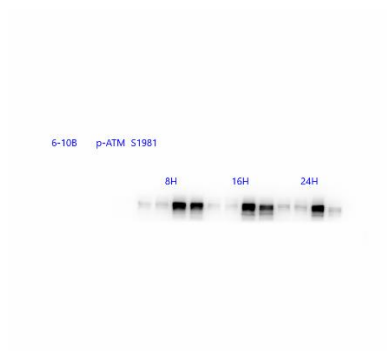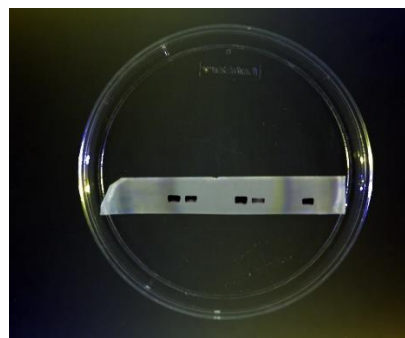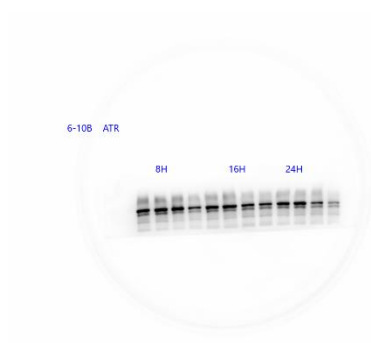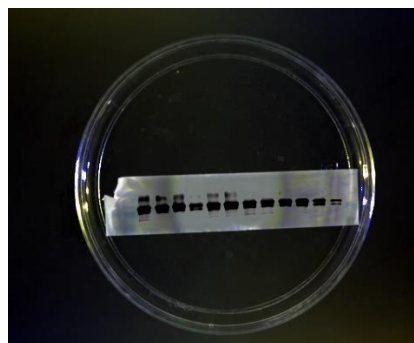

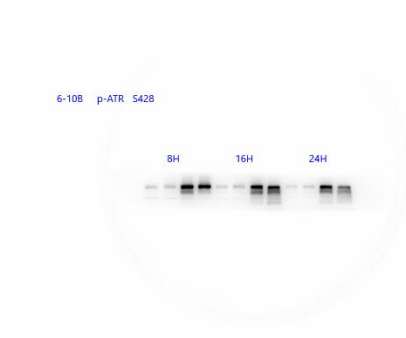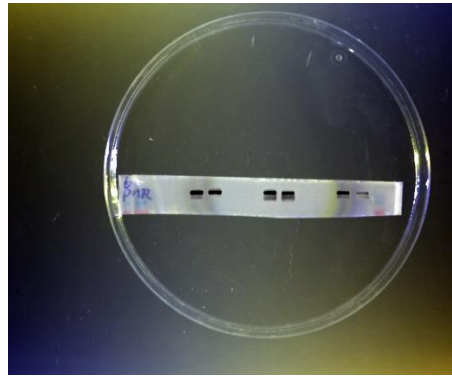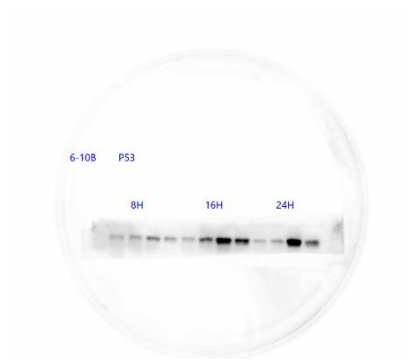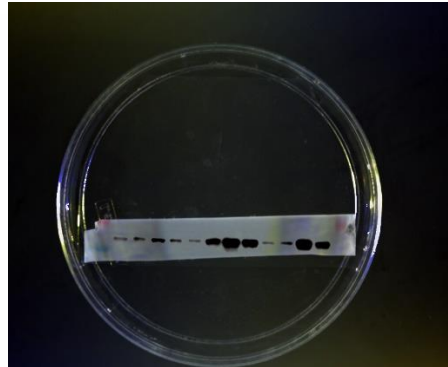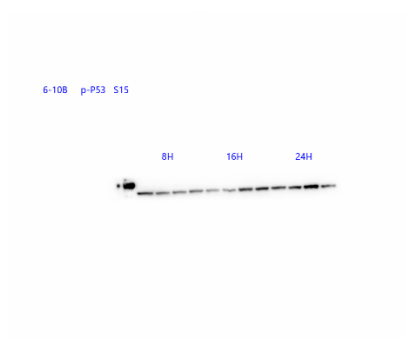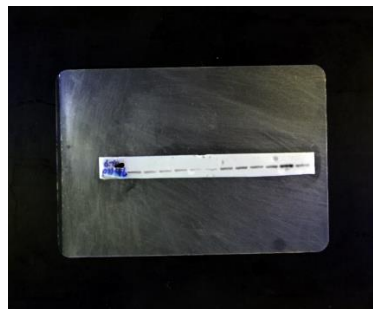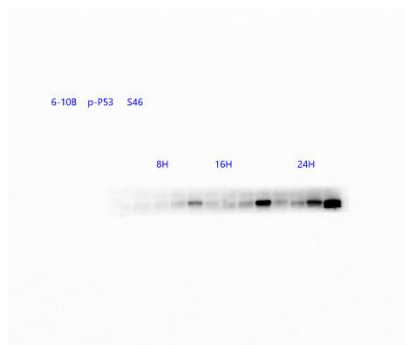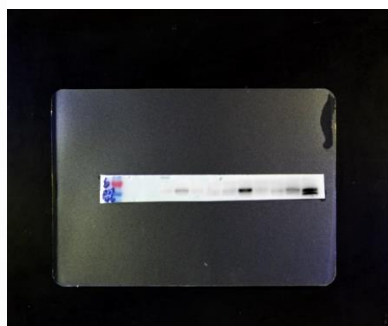

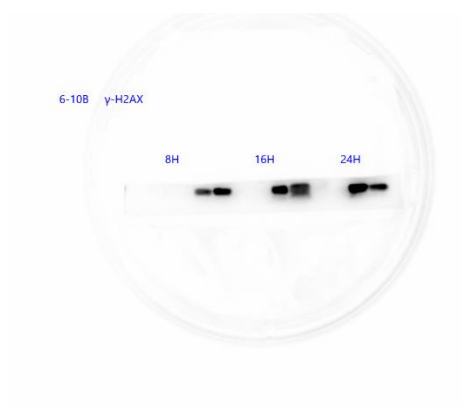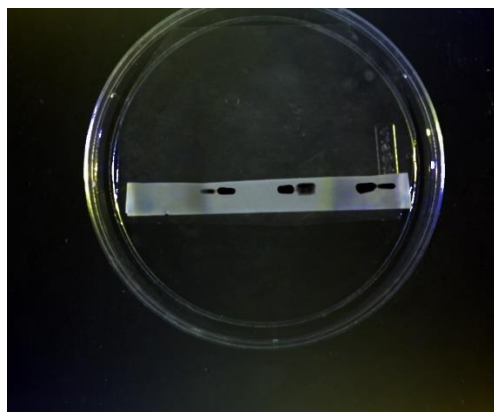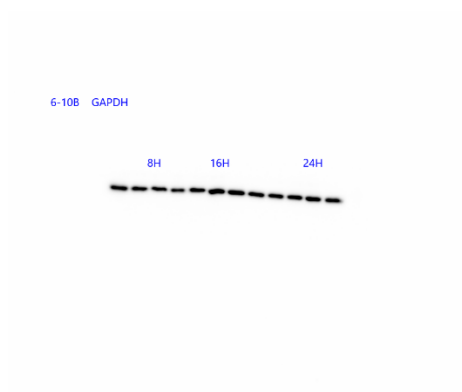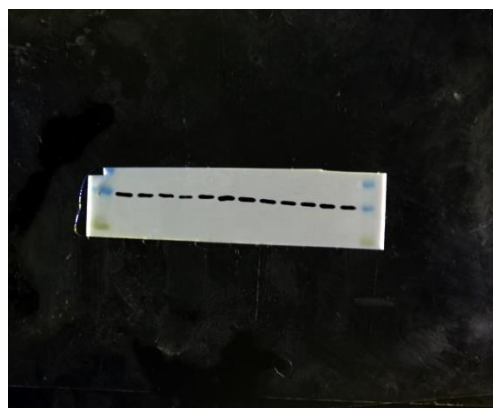

Supplement: S3 File — (PDF) [file pone.0329272.s003.pdf]
