# Supplementary material for: Premature termination of DNA Damage Repair by 3-Methyladenine potentiates cisplatin cytotoxicity in nasopharyngeal carcinoma cells
Source: PLoS One. 2025 Aug 4;20(8):e0329272. doi: 10.1371/journal.pone.0329272 (PMC12321125; doi:10.1371/journal.pone.0329272)
Supplement: S4 File — (PDF) [file pone.0329272.s004.pdf]

# Human tissue/cell STR test report

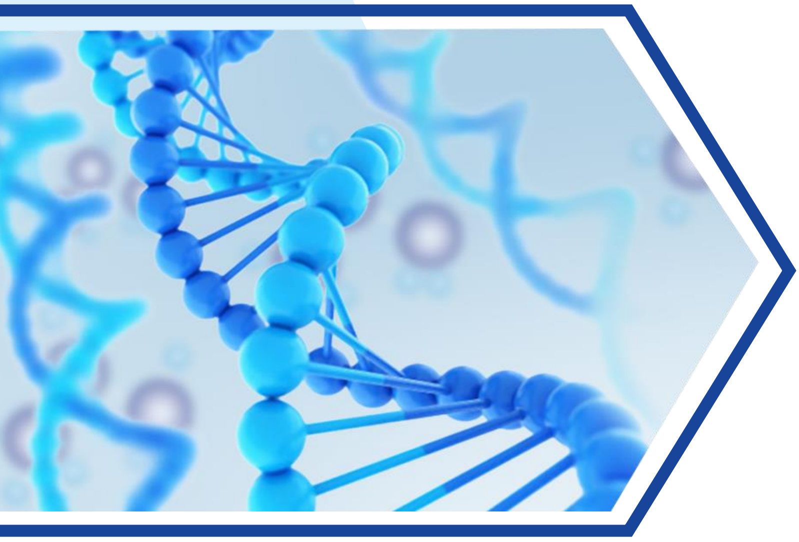

**Client:** **Changsha Abiwei Biotechnology Co.,Ltd.**

**Test indicators:** **STR genotyping**

**Report date:** **2025/05/12**

Huakejianlian Gene Technology (Beijing) Co., Ltd.

# Disclaimer

1. The result is only responsible for the samples submitted for inspection this time and objectively and truly reflects the outcome. It does not guarantee 100% compliance with the expectations of the client.
2. The content of this report and the information of the units involved may not be used for advertising, commercial exhibitions or other commercial activities without permission.
3. Without the permission of our company or without the official stamp of our company on the paper report, our company shall not be responsible for interpreting any changes, additions, or deletions to the content of this report.
4. If you have any objections to the result, please raise them within ten days.

# Sample

## Table 1. Sample Information

| Sample labeling | Test number | Sample type |
|---|---|---|
| 5-8F | WSE-HC-85 | Cell sedimentation |

**Date of Entrustment:** May 7, 2025

**Sample receipt date:** May 8, 2025

**Inspection date:** May 8, 2025

**The commissioning requirements:** STR typing test, comparison with the Cellosaurous database to confirm the identity of the cells, and at the same time monitor whether there is cross-contamination of cells of different types.

**Detection method:**

1. The amplification of 20 STR loci and 1 gender locus was conducted using a multiplex PCR amplification system (CELL STR ID®).
2. The PCR amplification products were analyzed using an ABI 3130xl DNA Analyzer (Applied Biosystems®).
3. The test results were analyzed using GeneMapper ID-X v1.5 (Applied Biosystems®) software.

**Testing basis:**

Refer to the cell authentication standards released by the International Cell Line Authentication Committee (ICLAC): For human cell authentication, the STR detection method is adopted. The old version (ASN-0002-2011) includes 8 STR gene loci, while the new version (ASN-0002-2022) recommends including 13 or more STR gene loci.

# Analysis and Explanation of Results

I. Test Results:

The results of both the negative and positive controls in the experiment were correct.

The genotyping results of STR loci and Amelogenin locus of this cell line are shown in the attached table, and the genotyping profiles are presented in the attached figures.

| Genetic Site | Cellosaurus | | | | Test sample | | | |
|---|---|---|---|---|---|---|---|---|
| (Locus) | Database name: 5-8F | | | | Sample Name: 5-8F | | | |
| Amelogenin | X | | | | X | | | |
| D5S818 | 11 | 12 | | | 11 | 12 | | |
| D13S317 | 10 | 12 | 13.3 | | 10 | 12 | 13.3 | |
| D7S820 | 10 | 12 | | | 10 | 12 | | |
| D16S539 | 9 | 10 | | | 9 | 10 | | |
| vWA | 14 | 16 | | | 14 | 16 | | |
| TH01 | 7 | 9 | | | 7 | 9 | | |
| TPOX | 8 | 9 | 12 | | 8 | 9 | 12 | |
| CSF1PO | 10 | 11 | | | 10 | 11 | | |
| The total number of matching peaks (excluding gender loci) | | | | | | | | 18 |
| The number of all peaks in the Cellosaurus database and the test samples | | | | | | | | 36 |
| Match degree (according to the Cellosaurus rule: 18 * 2 / (18 + 18)) | | | | | | | | 100% |

II. Analysis and Explanation:

The DNA amplification pattern of this cell was clear and the typing result was good.

III. Inspection Conclusion:

5-8F：

① The STR typing results of the DNA from this cell strain showed that trilateral genotypes were observed at the D6S1043, D13S317 and TPOX loci, while no multilateral genotypes were found at the other loci.

② 5-8F in the Cellosaurus database, cells with a 100% match (8 STR loci matched) to its cell typing were found, and the cell name is: 5-8F. The specific comparison results are shown in the attached figure.

③ At the request of the client, the homology between 5-8F cells and SUNE1 cells was compared. The results showed that the homology between 5-8F cells and SUNE1 cells was 97.3%. It can be basically determined that 5-8F cells and SUNE1 cells are related, that is, they are derived from a common ancestral cell.

(This result is only valid for the current sample.）

**Operator: Sun Yaomin** 孙瑶敏

**Reviewer: Zhu Lili** 朱莉莉

**Person in charge: Sun Shanlin** 孙山林

**Report date: 2025/05/12**

**Note:**

1. According to the cell STR identification standards established by the International Cell Line Authentication Committee (ICLAC), when the matching degree of cell lines is ≥ 80%, they are considered to be related, that is, derived from a common ancestral cell; when the matching degree is between 55% and 80%, further verification of their relatedness is required; when it is less than 55%, it indicates that the two are not related.
2. Since some cells in the database only have reference data for 9 loci (8 STR loci + 1 gender locus), during comparison, some results are based on the comparison of 8 STR loci. If there are reference data for more STR loci, the comparison results should be based on the new standard for STR identification of cells with more loci.
3. The effective peaks marked in the electrophoretic pattern are the true PCR bands; the small peaks and non-specific bands without typing results are ignored in the calculation.
4. The matching degree calculation is based on the default Cellosaurus calculation rule (2 * number of identical alleles / total number of alleles in the sample to be tested and the reference data), unless the client requests personalized requirements in advance.
5. If the client's cell samples are stem cells, immune cells, primary cells, etc., and have not been submitted to a public database, the STR data should be compared first with the STR data of the donor's own samples or earlier samples. If the laboratory has other cell lines in culture, the STR data can also be compared with the database to rule out contamination by cell lines with known STR data.
6. STR data comparison is by default based on the Cellosaurus database, which is currently the most authoritative and comprehensive source for STR data of cell lines. It integrates STR data of human and mouse cells from all databases or literature such as ATCC, DSMZ, JCRB, ECACC, COG, CBA, and KCLB. If there are any other specific comparison data requirements, the source of STR data for the sample to be tested must be specified in advance.
7. If a locus is typed with only one number, it indicates that the locus is homozygous; if there are two different numbers, it indicates that the locus is heterozygous. If there are multiple different numbers, it suggests that the locus has multiple alleles, indicating an abnormal diploid cell or cross-contamination of the cell.

**Appendix: Genotyping results of STR loci and Amelogenin locus of cell**

| 5-8F | | | | |
|---|---|---|---|---|
| Marker | Allele 1 | Allele 2 | Allele 3 | Allele 4 |
| D19S433 | 13 | | | |
| D5S818 | 11 | 12 | | |
| D21S11 | 27 | 30 | | |
| D18S51 | 13 | 16 | | |
| D6S1043 | 11 | 14 | 18 | |
| AMEL | X | | | |
| D3S1358 | 15 | 18 | | |
| D13S317 | 10 | 12 | 13. 3 | |
| D7S820 | 10 | 12 | | |
| D16S539 | 9 | 10 | | |
| CSF1PO | 10 | 11 | | |
| Penta D | 9 | 12 | | |
| D2S441 | 11 | 14 | | |
| vWA | 14 | 16 | | |
| D8S1179 | 12 | | | |
| TPOX | 8 | 9 | 12 | |
| Penta E | 17 | | | |
| TH01 | 7 | 9 | | |
| D12S391 | 20 | 21 | | |
| D2S1338 | 17 | 23 | | |
| FGA | 21 | | | |

# 人源组织/细胞 STR 检测报告

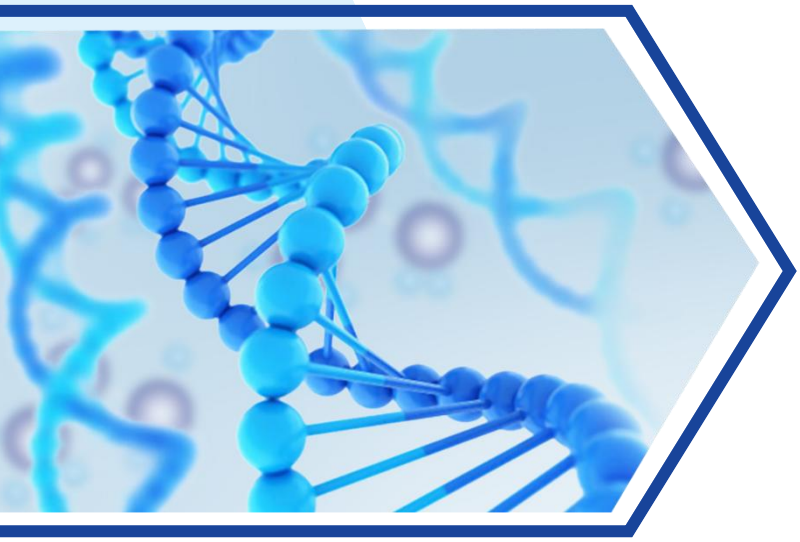

**委托方：长沙艾碧维生物科技有限公司**

**检测指标：STR 基因分型**

**报告日期：2025/05/12**

华科鉴联基因科技（北京）有限公司

# 申明

1. 结果仅对本次送检样品负责，并客观真实反映结果，不保证 100%符合委托方的预期。
2. 本报告内容及单位信息，未经许可，不得用于广告、商业展览和其他商业活动。
3. 本报告内容变更、增减未经本公司许可或无本公司盖章的纸质报告，本公司不承担解释责任。
4. 如对结果有异议，请于十日内提出。

# 表一、样本信息

| 样本标记 | 检测编号 | 样本类型 |
|---|---|---|
| 5-8F | WSE-HC-85 | 细胞沉淀 |

**委托日期:** 2025.05.07

**收样日期:** 2025.05.08

**检测日期:** 2025.05.08

**委托要求：** STR 分型检测，与 Cellosaurous 数据库比对确认细胞身份，同时监控是否存在种类不同细胞交叉污染现象。

**检测方法:**

1、用多重 PCR 复合扩增系统(CELL STR ID®)对 20 个 STR 位点和 1 个性别位点进行扩增；

2、PCR 扩增产物用 ABI 3130xl DNA Analyzer(Applied Biosystems®)进行分析；

3、检测结果用 GeneMapper ID-X v1.5 (Applied Biosystems®)软件进行分析。

**检测依据:**

参考国际细胞鉴定委员会（ICLAC）发布的细胞鉴定标准：人源细胞鉴定采用 STR 检测方法。其中旧版（ASN-0002-2011）：包含 8 个 STR 基因座位点。新版（ASN-0002-2022）建议包含 13 个及以上 STR 基因座位点。

# 结果分析说明

一、检验结果：

实验中阴性及阳性对照结果均正确。

该细胞株的 STR 位点和 Amelogenin 位点的基因分型结果见附表，分型图谱见附图。

| Genetic Site | Cellosaurus | | | | 检测样品 | | | |
|---|---|---|---|---|---|---|---|---|
| (Locus) | 数据库名称：5-8F | | | | 样品名称：5-8F | | | |
| Amelogenin | X | | | | X | | | |
| D5S818 | 11 | 12 | | | 11 | 12 | | |
| D13S317 | 10 | 12 | 13.3 | | 10 | 12 | 13.3 | |
| D7S820 | 10 | 12 | | | 10 | 12 | | |
| D16S539 | 9 | 10 | | | 9 | 10 | | |
| vWA | 14 | 16 | | | 14 | 16 | | |
| TH01 | 7 | 9 | | | 7 | 9 | | |
| TPOX | 8 | 9 | 12 | | 8 | 9 | 12 | |
| CSF1PO | 10 | 11 | | | 10 | 11 | | |
| 所有匹配峰的个数(不含性别位点） | | | | | | | | 18 |
| Cellosaurus 数据库和检测样所有峰的个数 | | | | | | | | 36 |
| 匹配度（按 Cellosaurus 的规则：18*2/(18+18)） | | | | | | | | 100% |

## 二、分析说明：

该细胞 DNA 扩增后图谱清晰，分型结果良好。

## 三、检验结论：

5-8F：①该株细胞 DNA 进行细胞 STR 分型结果显示，D6S1043,D13S317,TPOX 基因座出现三等位基因现象，其余各基因座均未出现多等位基因现象。

②5-8F 在 Cellosaurus 数据库中找到与其细胞分型 100%相匹配（8 个 STR 位点匹配）的细胞，细胞名为：5-8F。具体比对结果见附图。

③应客户要求将 5-8F 细胞与 SUNE1 细胞的同源性进行比对，结果显示 5-8F 细胞与 SUNE1 细胞的同源性有 97.3% 。可基本确定 5-8F 细胞与 SUNE1 具有相关性，即衍生于共同的祖先细胞。

（此结果仅对本次检材负责）

**操作人: 孙瑶敏** 孙瑶敏

**审核人: 朱莉莉** 朱莉莉

**负责人: 孙山林** 孙山林

**报告日期:** 2025/05/12

备注：

1. 根据国际细胞鉴定委员会(ICLAC)制定的细胞 STR 鉴定标准，细胞系的匹配度≥80% 时，认为它们具有相关性，即衍生于共同的祖先细胞；匹配度在 55% 至 80% 之间，需要进一步验证相关性；小于 55%，表明两者不具有相关性。
2. 因数据库有的细胞只有 9 个位点（8 个 STR+1 个性别位点）的参考数据，故比对时，有的结果是 8 个 STR 位点比对；如有更多位点的 STR 参比数据，参考新版细胞 STR 鉴定标准，则采用更多位点的参比数据比对结果。
3. 图谱标注分型的有效峰为真实的 PCR 条带;未标注分型结果的小峰和非特异性条带在计算中忽略不计。
4. 本匹配度计算默认依照 Cellosaurus 计算规则(2*相同等位基因数/待检样本等位基因数与参比数据等位基因数总和)，若有个性化需求委托方可提前提出。
5. 委托方细胞样本若为干细胞，免疫细胞，原代细胞等细胞，且未提交至公开数据库，STR 数据优先与自身 donor 样本或早期样本 STR 数据比对。如实验室有其它细胞系培养时，也可与数据库比对，排除是否被已知 STR 数据的细胞系污染。
6. **STR 数据比对默认 Cellosaurus 数据库，该数据库是目前最权威最全面的细胞系 STR 数据库比对来源，集合了 ATCC，DSMZ，JCRB、ECACC、COG、CBA、KCLB 等所有数据库或文献发布的人源和小鼠细胞 STR 数据，若有其它个性比对数据要求需提前注明被检样本的 STR 数据比对来源。**
7. 基因座分型只有一个数字，表示该基因座为纯合子；基因座有两个不同数字，表示该基因座为杂合子。若有多个不同数字，表示该基因座有多等位基因，提示非正常二倍体细胞或该细胞被交叉污染。

附表：细胞 5-8F 的 STR 位点和 Amelogenin 位点的基因分型结果

| 5-8F | | | | |
|---|---|---|---|---|
| Marker | Allele 1 | Allele 2 | Allele 3 | Allele 4 |
| D19S433 | 13 | | | |
| D5S818 | 11 | 12 | | |
| D21S11 | 27 | 30 | | |
| D18S51 | 13 | 16 | | |
| D6S1043 | 11 | 14 | 18 | |
| AMEL | X | | | |
| D3S1358 | 15 | 18 | | |
| D13S317 | 10 | 12 | 13.3 | |
| D7S820 | 10 | 12 | | |
| D16S539 | 9 | 10 | | |
| CSF1PO | 10 | 11 | | |
| Penta D | 9 | 12 | | |
| D2S441 | 11 | 14 | | |
| vWA | 14 | 16 | | |
| D8S1179 | 12 | | | |
| TPOX | 8 | 9 | 12 | |
| Penta E | 17 | | | |
| TH01 | 7 | 9 | | |
| D12S391 | 20 | 21 | | |
| D2S1338 | 17 | 23 | | |
| FGA | 21 | | | |
